# Supplementary material for: Distinct Bomanins at the Drosophila 55C locus function in resistance and resilience to infections
Source: EMBO Rep. 2026 Jan 9;27(3):629–53. doi: 10.1038/s44319-025-00559-6 (PMC12894722; doi:10.1038/s44319-025-00559-6)
Supplement: Supplementary file 1 — Appendix [file 44319_2025_559_MOESM1_ESM.pdf]

APPENDIX

Distinct Bomanins at the **Drosophila 55C** *locus function in resistance and resilience to infections*

Appendix Table of Contents

Appendix Figure S1. 3

Appendix Figure S2. 4

Appendix Figure S3 6

Appendix Figure S4 8

Appendix Figure S5 10

Appendix Figure S6 11

Appendix Figure S7 12

Appendix Figure S8 14

Appendix Figure S9. 16

Appendix Figure S10 18

Appendix Figure S11 20

Appendix Figure S12 22

Appendix Table S1 25

Appendix Table S2 23

Appendix Table S3 24

Appendix Table S4 27

Appendix Figures

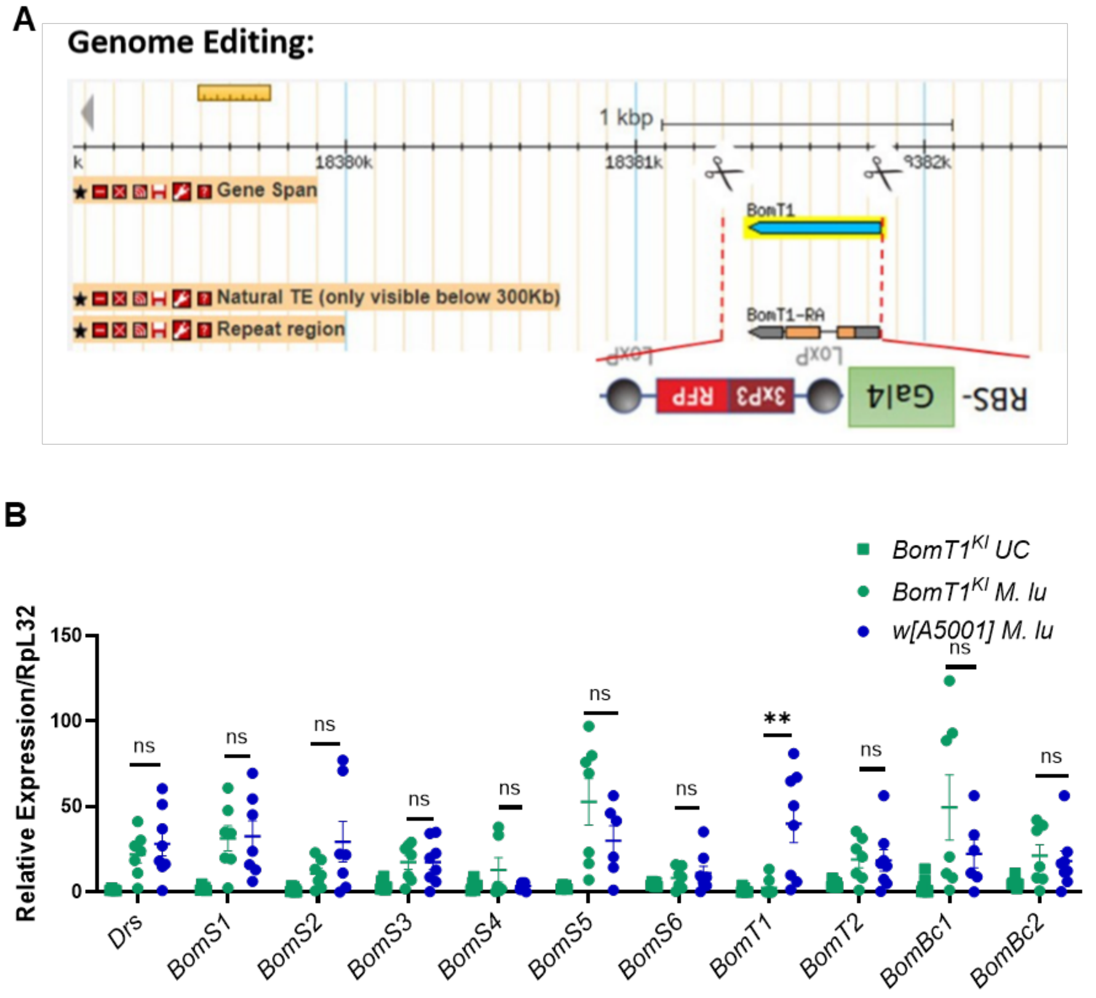

Appendix Figure S1. ***BomT1*^{KI} genetic information**

(A) Schematic view of the transgenic sequence that replaces *BomT1* coding sequences to generate the *BomT1-Gal4* knock-in (*BomT1*^{KI}) mutant. The *BomT1* ORF was replaced with the Gal4 sequence preceded by optimized Kozak sequences: RBS (ribosome-binding site); 3-P3-RFP is used as an eye fluorescent marker for the presence of the transgene.

(B) The steady-state transcript levels of *Drosomycin* and *Bomanin* genes located at 55C site in *BomT1*^{KI} flies 24 hours post *M. luteus* (*M. lu*) injection.

Data information: (B) Three experiments were performed at different times and each experiment used biological triplicates of five flies in parallel. The pooled data are presented as means ± SEM. Statistical analysis was performed using the Mann-Whitney test between *BomT1*^{KI} and *w* [A5001] *M. luteus*-challenged flies. UC: unchallenged. **: $p<0.01$; ns: not significant.

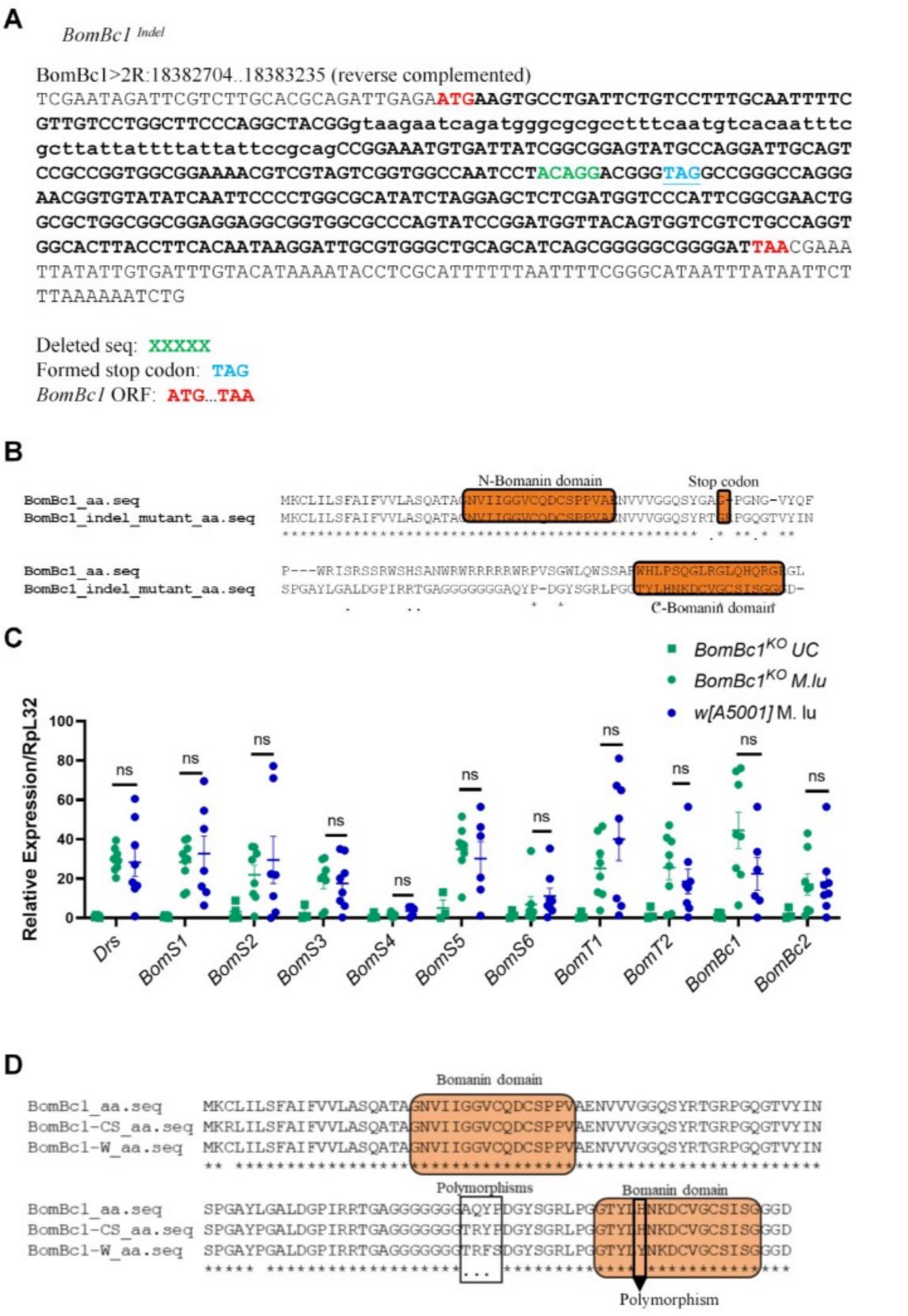

Appendix FigureS2. ***BomBc1*** ^{indel} **genetic information and distinct polymorphisms of *BomBc1* amino acid sequence for overexpression in transgenic flies**

(A) Genomic sequence of the *BomBc1*^{indel} mutant. Notes: The start and stop codons of the wild-type gene are indicated in red; this applies also to other Appendix figure legends. The novel stop codon formed as a result of the indel mutation is shown in blue.
(B) Amino acid sequence prediction of the *BomBc1*^{indel} mutant. 5 nucleotides (nts) are deleted and give rise to a stop codon, thus only the second Bomanin domain in the C-terminal was removed.
(C) The transcription level of *BomBc1*, *Drosomycin* and other *Bom* genes of *BomBc1*^{indel} post *M. luteus* injection at 24 hours.
(D) Translation sequence of transgenic flies in *BomBc1*^{w1118} and *BomBc1*^{CS} compared with *BomBc1* translation sequence from Flybase (https://flybase.org/). A polymorphism is found in four amino acids which are 'AQYP' in Flybase, 'TRYP' in *BomBc1*^{CS}, and 'TRFS' in *BomBc1*^{w1118}. There is an additional polymorphism for *BomBc1*^{w1118} in the second Bomanin domain as indicated.
Data information: (C) Three experiments were performed at different times and each experiment used biological triplicates of five flies in parallel. The pooled data are presented as means ± SEM. Statistical analysis was performed using the Mann-Whitney test between *BomBc1*^{KO} and *w* [A5001] *M. luteus* (*M. lu*)-challenged flies. ns: not significant. UC: unchallenged. ns, no significant difference.

A

BomS2(IM2, CG18106) >2R:18386602..18387030
CCAAGAGCATCAGTTGAATTCAATCGATTGCTTGTGCATTTAGCAAATCAAAGCCAC
AACAACCAAACCAGAATCAATATGAAGTTCTTCTCAGTCGTCACCGTCTTTGTGTTC
GGTCTGCTGGCTCTGGCCAACGgttagtaatactatattttatagctatttgattactttataaattatttatttcttccgcagC
TGTTCCCCTGTCGCCCGATCCAGGAAATGTGGTAATCAACGGGGACTGCAAATACT
GCAATGTGCACGGTGGAAAGTAGGAAAGTAGGAAAGTAGGAAAGTACTCGCCTTA
ATTTCGAAGATGGGCCAAAACTTACCTCAAATCCAAAGCACCATATTTATACTCTCA
CTCTTGTACTAAAATGAAAACTAGTAGTAAAAAATACATCGCCAATTACAAATAAA
ATGGTGAAAAAAC

BomS2$^{\Delta KO36}$ knock out sequence

BomS2$^{\Delta KO6}$ knock out sequence

BomS2 ORF ATG…GGTGGAAAGTAG

Intron sequence: gttagtaatactatattttatagctatttgattactttataaattatttatttcttccgcag

B

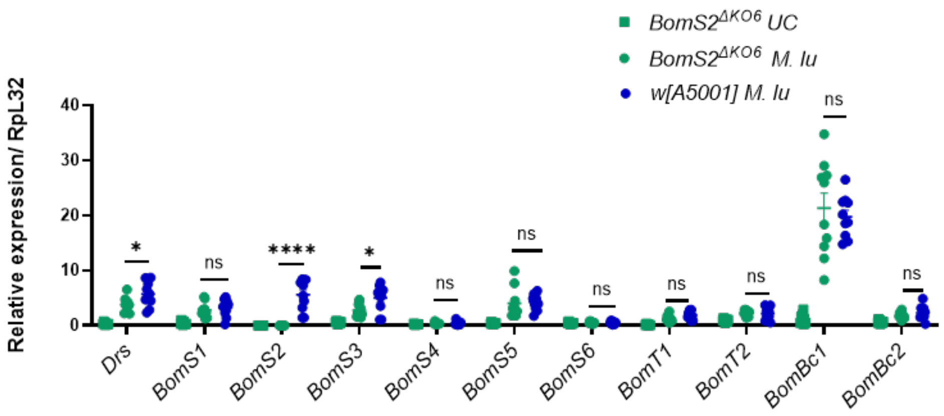

C

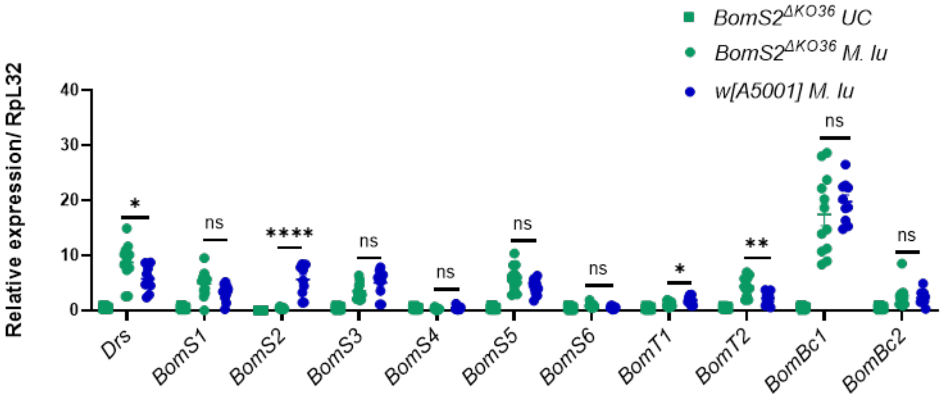

Appendix Figure S3. Genetic information on two *BomS2* knock out mutants

(A) *BomS2*$^{\Delta KO6}$, and *BomS2*$^{\Delta KO36}$ are two null deletion mutants generated using two gRNAs at the same time. 135nts (counting from ATG, which was not removed in *BomS2*$^{\Delta KO36}$) were deleted in *BomS2*$^{\Delta KO6}$, and only 12nts (sequence in blue,

GGTGGAAAGTAG) were left in *BomS2*$^{\Delta KO36}$ mutants. ORF: open reading frame.

(B-C) The transcription level of *BomS2*, *Drosomycin* and other *Bom* genes of *BomS2*$^{\Delta KO6}$ (B) and *BomS2*$^{\Delta KO36}$ (C) post *M. luteus* injection at 24 hours.

Data information: (B-C) Three experiments were performed at different times and each experiment used biological triplicates of five flies in parallel. The pooled data are presented as means ± SEM. Statistical analysis was performed using the Mann-Whitney test between *BomS2* mutants and *w* [A5001] *M. luteus* (*M. lu*)-challenged flies. *: p<0.05; **: p<0.01; ****: p<0.0001; ns, no significant difference ns: not significant. UC: unchallenged.

A *BomT2*^{KI312}

>2R:18388995..18389443

CAATTATATATATAATATAGCAAATACTATATGTACTATGGCATATAAAAAGCAG
AGCACTCGCACTTTGGCAGTCAGATTTATTCGAAATCCCCAAGGAAAAACATGAA
AGCTCTTCAAGTCGCCGGAACTTTGATGCTGCTTTTCTGCCTGCTGGCAGCTGTTA
ATGgtaattatttaatgaaataataaaatatatatatgatatttttagctaatcatttccttattgcagCTACGCCGGGACAAG
TGTATATCAATGGGAAATGCATTGACTGCAATAAGCCTGATAATGATCCGGGAAT
TATAATTCCTCCAGACCATAAATCAGCTGGATCCATGTCTTACACACTCACATCTG
GAGCCATCTTCTTTGGAATTATATATCATATATTCAGTTAAATTACTATGTAATCA
ATAAGTTAATAAAATAATATCTTTTATTTCTCaaaaaaaaaccttaagcttgtgacaaattgcgaaattgc
gaa

Replaced sequence: XXXXXX
***BomT2* ORF:** **ATG…TAA**
Intron sequence: gtaattatttaatgaaataataaaatatatatatgatatttttagctaatcatttccttattgcag

B *BomT2*^{KO259}

>2R:18388995..18389443

CAATTATATATATAATATAGCAAATACTATATGTACTATGGCATATAAAAAGCAG
AGCACTCGCACTTTGGCAGTCAGATTTATTCGAAATCCCCAAGGAAAAACATGAA
AGCTCTTCAAGTCGCCGGAACTTTGATGCTGCTTTTCTGCCTGCTGGCAGCTGTTA
ATGgtaattatttaatgaaataataaaatatatatatgatatttttagctaatcatttccttattgcagCTACGCCGGGACAAG
TGTATATCAATGGGAAATGCATTGACTGCAATAAGCCTGATAATGATCCGGGAAT
TATAATTCCTCCAGACCATAAATCAGCTGGATCCATGTCTTACACACTCACATCTG
GAGCCATCTTCTTTGGAATTATATATCATATATTCAGTTAAATTACTATGTAATCA
ATAAGTTAATAAAATAATATCTTTTATTTCTCaaaaaaaaaccttaagcttgtgacaaattgcgaaattgc
gaa

Knock out sequence: **XXXXXX**
***BomT2* ORF:** **ATG…TAA**

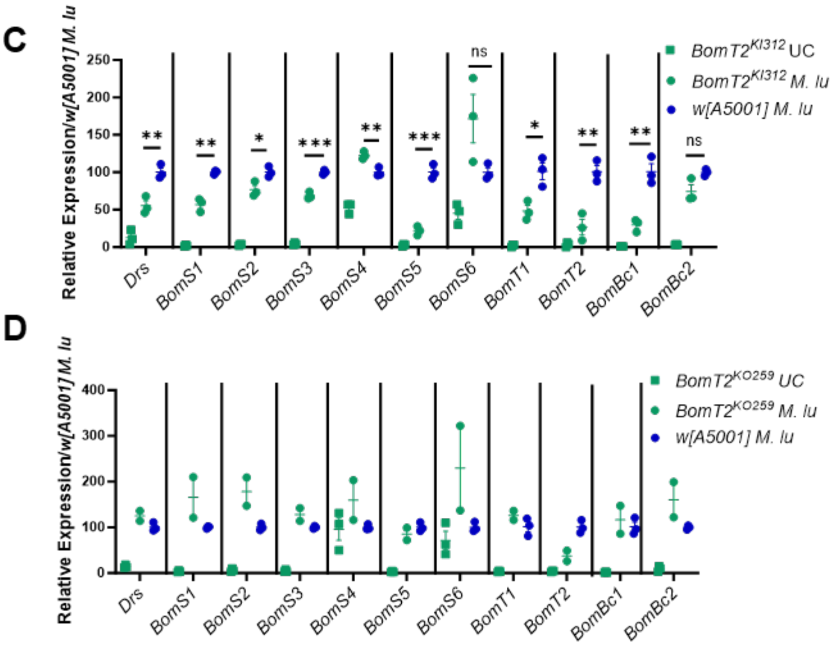

Appendix Figure S4. **Genetic information on two *BomT2* mutants**

(A) *BomT2-mCherry* knock-in sequence (*BomT2*^{KI312}). The sequence underlined and in grey has been replaced by the *mCherry* coding sequence (not shown). ORF: open

reading frame.

(B) *BomT2* knock-out sequence (*BomT2*^{KO259}). The sequence in green had been deleted. The start and stop codons from the *BomT2* coding sequence (ORF) are indicated in red. The sequences shown in blue remain from the original gene.

(C-D): Steady-state transcripts of 55C *Bom* genes measured by RT-qPCR.

Data information: (C-D) Statistical analysis has been performed using the unpaired t-test in one experiment with biological triplicates of 5 flies. *: $p<0.05$; **: $p<0.01$; ***: $p<0.001$; ns, no significant difference.

A

BomT2 (IM28, CG16836) >2R:18388995..18389443

BomT2$^{\Delta tail}$

TAATTATATATATAATATAGCAAATACTATATGTACTATGGCATATAAAAAGCAGA
GCACTCGCACTTTGGCAGTCAGATTTATTCGAAATCCCCAAGGAAAAACATGAAAG
CTCTTCAAGTCGCCGGAACTTTGATGCTGCTTTTCTGCCTGCTGGCAGCTGTTAATG
gtaattatttaatgaaataataaaatatatatatgatatttttagctaatcatttccttattgcagCTACGCCGGGACAAGTGTA
TATCAATGGGAAATGCATTGACTGCAATAAGCCTGATAATGATCCGGGAATTATAA
TTCCTCCAGACCATAAATCAGCTGGATCCATGTCTTACACACTCACATCTGGAGCCA
TCTTCTTTGGAATTATATATCATATATTCAGTTAAATTACTATGTAATCAATAAGTT
AATAAAATAATATCTTTTATTTCTC

knock out nucleotides : TGATAATGA

***BomT2* ORF: ATG…TAA**

B

```
                                               Bomanin domain     Stop codon
BomT2.seq              MKALQVAGTLMLLFCLLAAVNATPGQVYINGKCIDCNKPDNDPGIIIPPDHKSAGSMSYT
BomT2_Tail_mutant.seq  MKALQVAGTLMLLFCLLAAVNATPGQVYINGKCIDCNKPFRE--LFLQTINQLDPCLTHS
                       ***************************************  .  . .   ..   ... .

BomT2.seq              -LTSGAIFFGIIYHIFS
BomT2_Tail_mutant.seq  HLEPSSLELYIIYSV--
                        *   ..   *** .
```

C

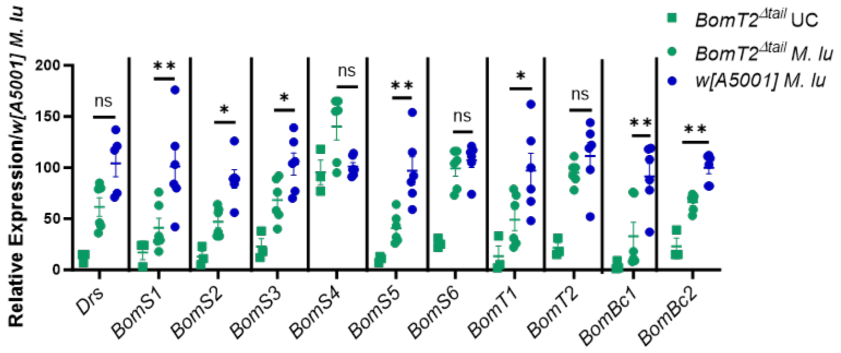

Appendix Figure S5. ***BomT2***$^{\Delta tail}$ **genetic information**

(A) *BomT2* nucleotide sequence in *BomT2*$^{\Delta tail}$ mutant consists of a deletion of two and five nucleotides separated by a two-nucleotide interval (TG--AATGA) in the coding sequence (ORF), indicated in green.

(B) Amino acid alignment results of *BomT2*$^{\Delta tail}$ flies compared with the *BomT2* wild-type sequence. The mutant predicted sequence formed a premature stop codon, and the C-terminal tail sequence was deleted. However, the *Bomanin* domain is still present in the mutant line. ORF: open reading frame.

(C) The expression level of 55C *Bomanin* genes was measured by RT-qPCR in *BomT2*$^{\Delta tail}$ mutants after *M. luteus* injection at 24 hours.

Data information: (C) Three experiments were performed at different times and each experiment used biological triplicates of five flies in parallel. The data are presented as means ± SEM and analyzed using the Mann-Whitney test. *: $p<0.05$; **: $p<0.01$; ns, no significant difference.

Figure S6

A

BomS5$^{\Delta KO}$

>2R:18389959..18390242

ATCAGTTTCATTTCAACCGTTGCCAACAAT**ATG**AAGTGGATGTCCTTGGTCTTTCTATGC
GGTCTGCTCGCCATGGCAGTGGgtgagtatctataagattcatacatccttaaccagtatcctaattatttttctactattactatag
CTTCTCCGTTAAATCCGGGTAATGTCATTATCAATGGAGATTGCCGTCATTGTAATGTTC
GCGGAGGC**TAA**ATTGGAGTTATAAAACTTTGATGTTTAGTAACTACAATAAAACTTATG
AAAAATACAGATTGTGC

Knock out sequence: XXXXXX
***BomS5* ORF: ATG…TAA**
Intron sequence: gtgagtatctataagattcatacatccttaaccagtatcctaattatttttctactattactatag

B

```
                                                       Bomanin domain
BomS5_KO_mutant.seq MKWMSLVFLCG------------------------------
BomS5.seq           MKWMSLVFLCGLLAMAVASPLNRGNVIINGDCRHCNVRGG
                    ***********
```

C

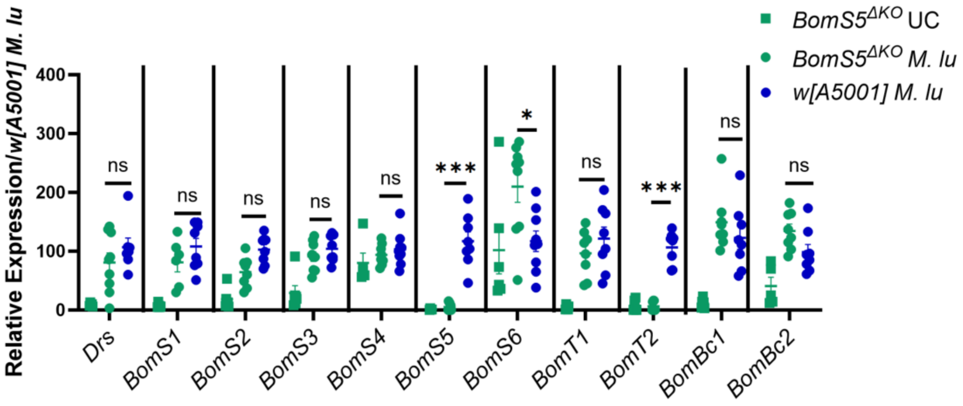

Appendix Figure S6. ***BomS5$^{\Delta KO}$* genetic information**

(A) The BomS5 mutation *BomS5$^{\Delta KO}$* consists in a 153-nucleotides deletion inside the coding sequence (ORF), indicated in green.

(B) Translation sequence alignment of *BomS5$^{\Delta KO}$*. Peptide sequence alignment between BomS5$^{\Delta KO}$ and the wild-type BomS5.

(C) Expression level of other 55C locus *Bom* genes measured by RT-qPCR in *BomS5$^{\Delta KO}$* mutants after *M. luteus* injection at 24 hours.

Data information: (C) Three experiments were performed at different times and each experiment used biological triplicates of five flies in parallel. The pooled data are presented as means ± SEM and analyzed using the Mann-Whitney test. *: $p<0.05$; ***: $p<0.001$; ns, no significant difference.

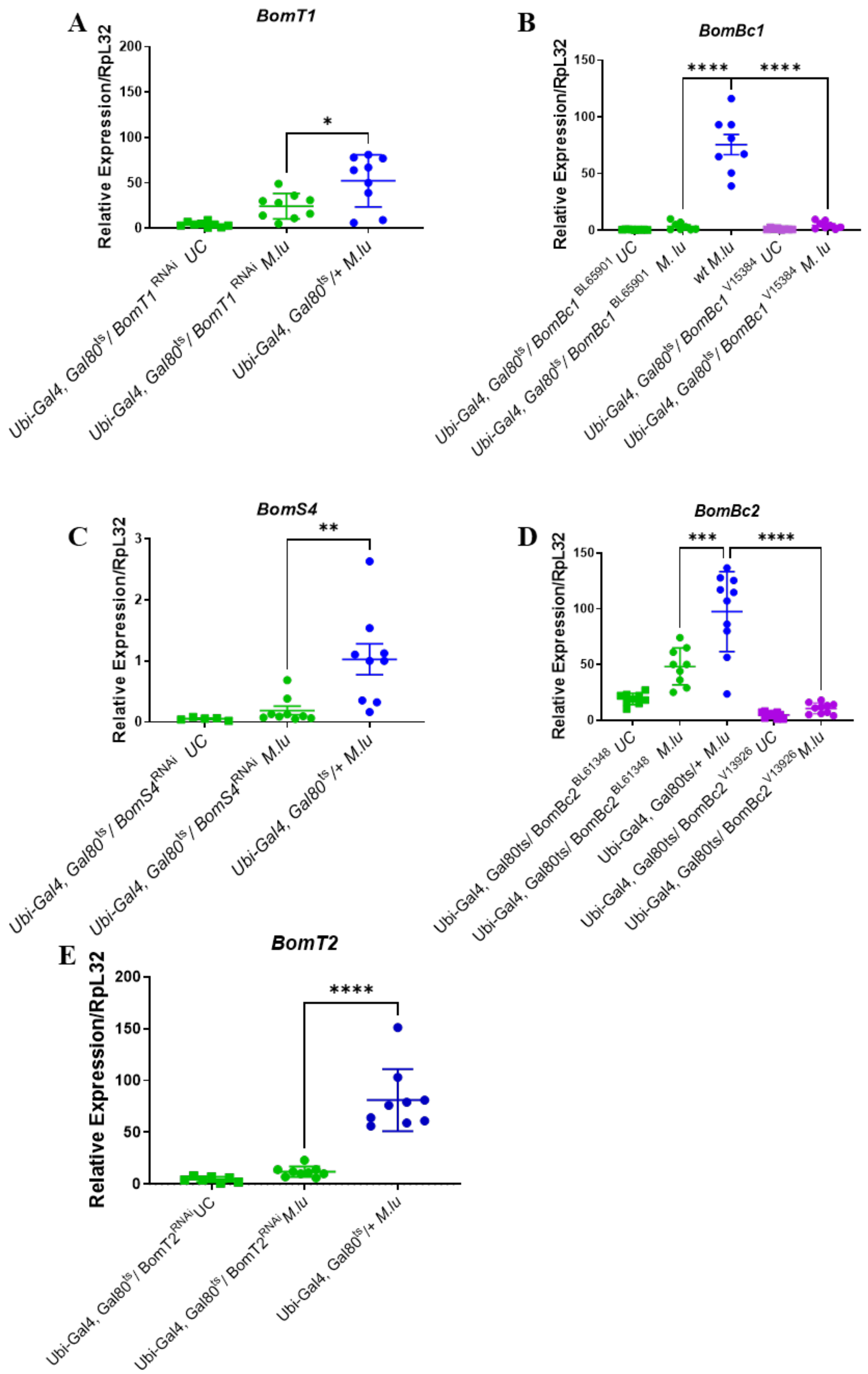

Appendix Figure S7. The efficiency of RNAi for *BomT1, BomBc1, BomS4, BomBc2* and *BomT2* knock down flies.

(A-E) Knock-down efficiency of *BomT1* (A), two *BomBc1* (B), *BomS4* (C), two *BomBc2* (D), and *BomT1* (E) knock-down RNAi fly lines measured by RT-qPCR.

Data information: (A-E) Three experiments were performed at different times and each experiment used biological triplicates of five flies in parallel. The pooled data are presented as means ± SEM and analyzed using the Mann-Whitney test (A, C, and E) test or one way ANOVA and Tukey's post-hoc test (B and D); *: $p<0.05$; **: $p<0.01$; ***: $p<0.001$; ****: $p<0.0001$.

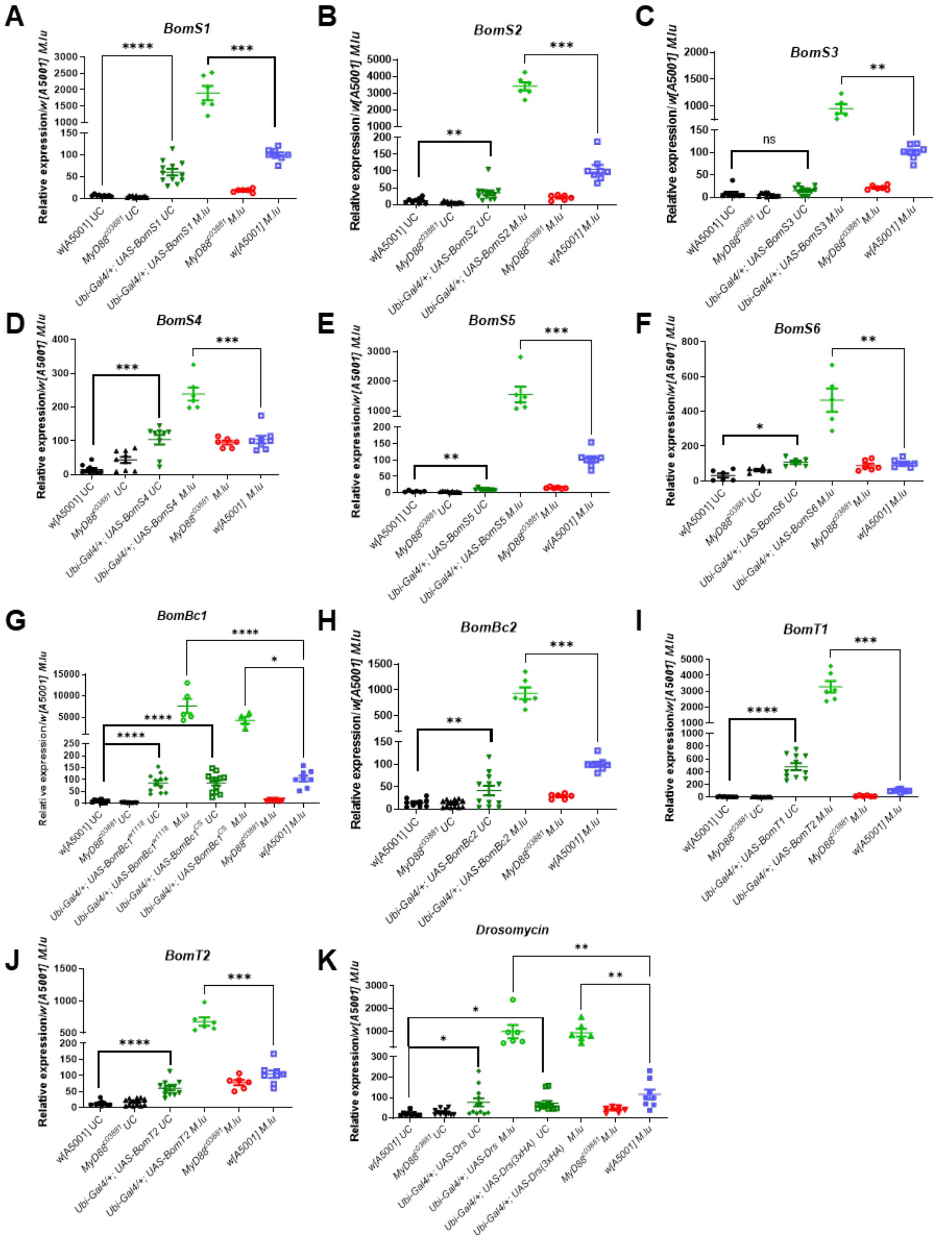

Appendix Figure S8. The efficiency of overexpression of single *Bomanin* genes in wild-type fly background.

(A-K) Expression level of single *Bomanin* gene overexpressing flies in a wild-type background by RT-qPCR. The data have been normalized to the level of induction of the *Bomanin* gene induced by *M. luteus* (*M. lu*) 24h after challenge in *w*[A5001] wild-type flies taken to be 100. Please, note that for many genes, the level of expression

reached by the overexpression approach in the absence of immune challenge is not as strong as that reached upon challenging wild-type flies with *M. luteus*, one exception being *BomT1*. The genotype of transgenic flies is: *w; Ubi-Gal4, Gal80ts/+; UAS-Bom/+*.

Data information: (A-K) Three experiments were performed at different times and each experiment used biological triplicates of five flies in parallel. The data are presented as means ± SEM and analyzed using ANOVA (one-way) with Tukey's post hoc test (*BomBc1* & *Drosomycin*) and by Mann-Whitney test for the others. *: $p<0.05$; **: $p<0.01$; ***: $p<0.001$; ****: $p<0.0001$; ns, no significant difference. UC: unchallenged. *BomBc1*^{CS}: Canton-S polymorphism isoform of the *BomBc1* gene.

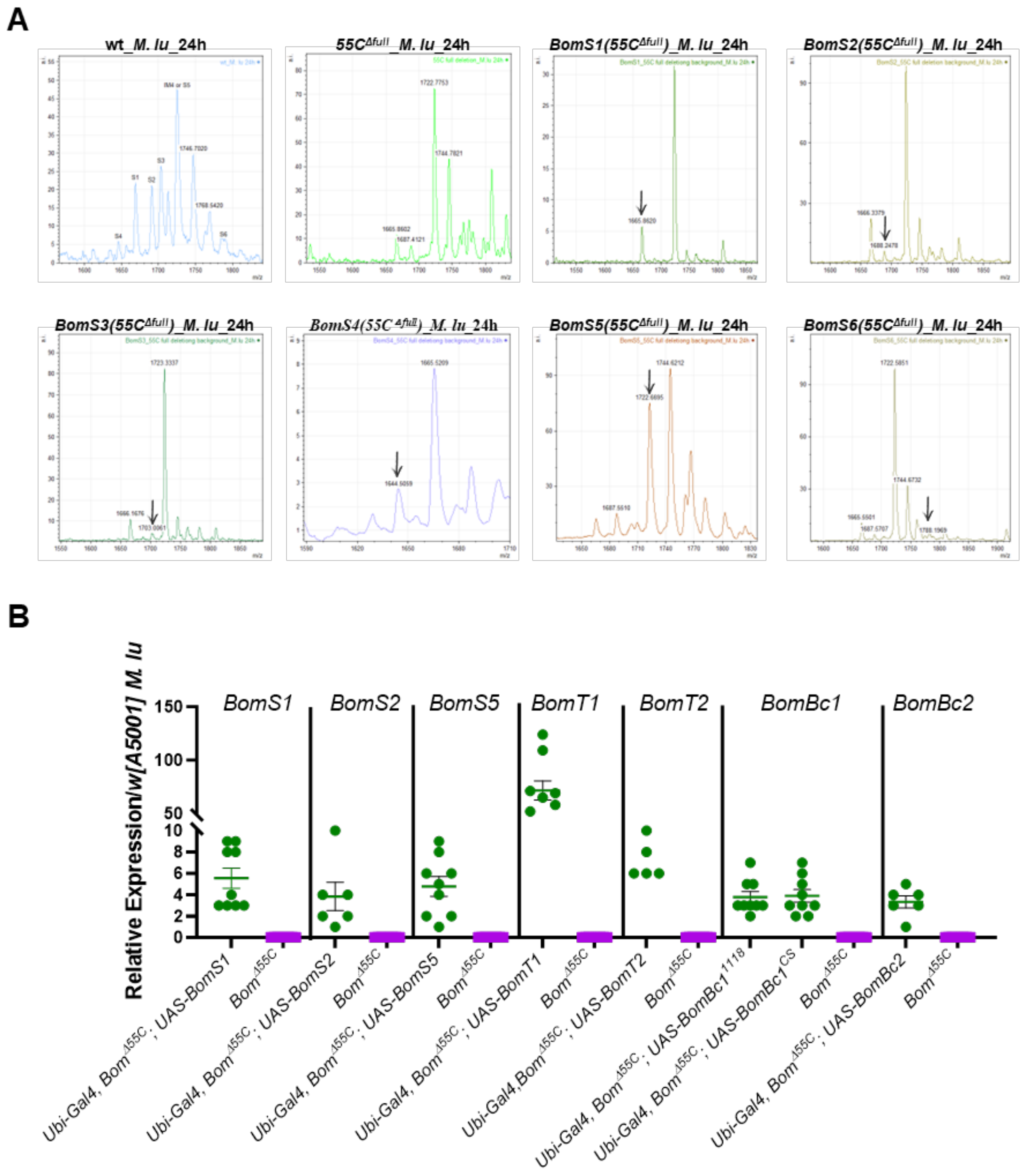

Appendix Figure S9. **Overexpression of single *Bomanin* genes in *Bom^{Δ55C}* fly background at the translational and transcriptional levels.**

(A) Analysis of the expression of some BomS peptides by MALDI-TOF mass spectrometry. More than five individual flies were tested for each overexpressed *BomS* gene, wild-type (wt: *w*[A5001]) and *Bom^{Δ55C}*. Note the presence of background peaks corresponding to the expected size for BomS1 and BomS2 in the *Bom^{Δ55C}* mutant background. This is also the case for BomS5, the molecular weight of which is very close to that of DIM4/Daisho1 and that can be actually observed in *Daisho* mutants (Cohen *et al*., Frontiers Immunol., 2020). The presence of these peaks does not allow drawing firm conclusions with respect to the actual presence of the overexpressed

BomS1, BomS2, and BomS5 peptides. An arrow in each panel indicates the expected position (molecular weight) of the overexpressed peptide.
(B) Expression level of *BomS1, BomS2*, Bom*S5*, *BomTs*, and the two *BomBcs* isoforms overexpressing flies in the *Bom*$^{\Delta 55C}$ background by RT-qPCR.
Data information: (B) Three experiments were performed at different times and each experiment used biological duplicates of five flies in parallel. The pooled data are presented as means ± SEM. No amplification was observed in the *Bom*$^{\Delta 55C}$ deletion mutant.

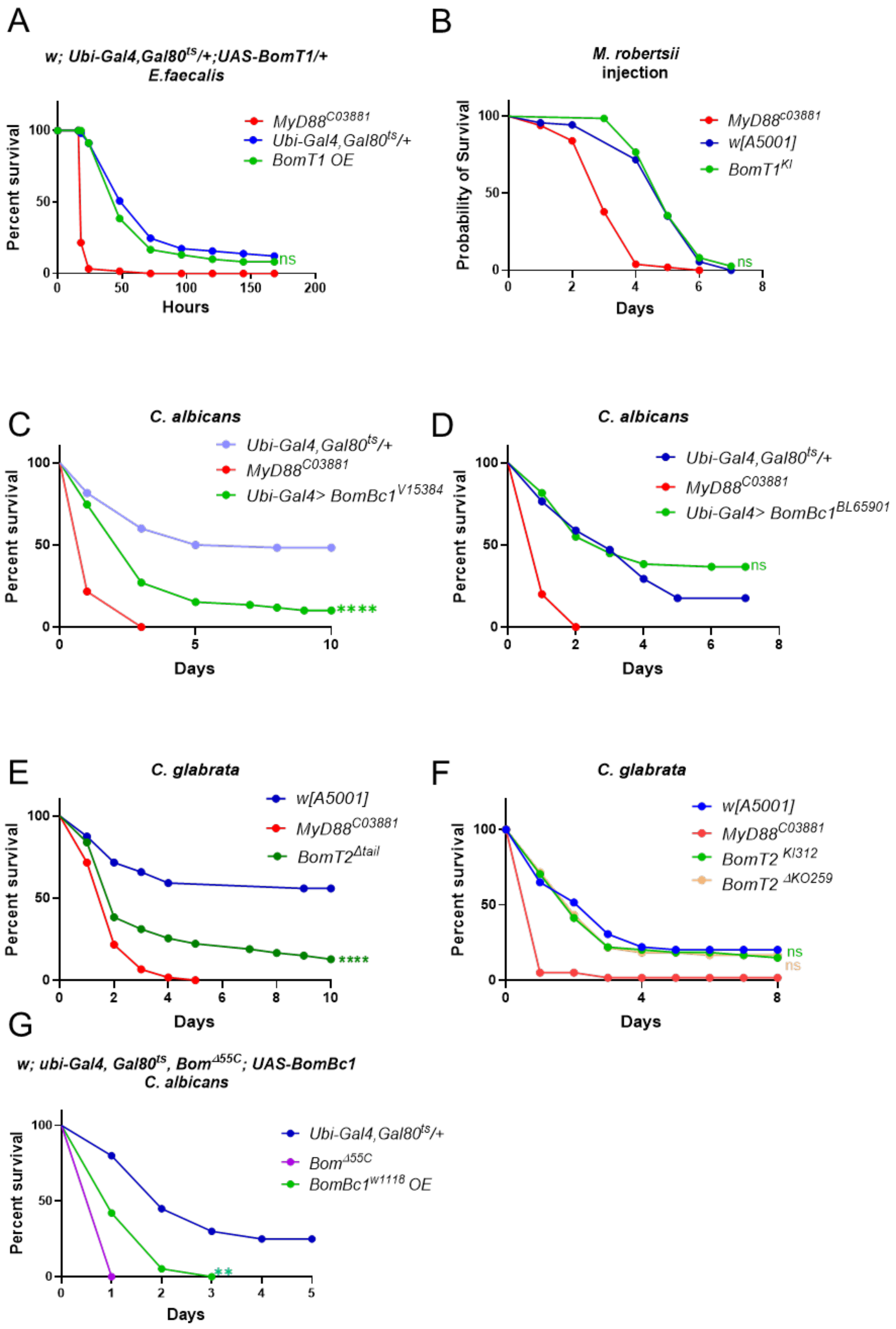

Appendix Figure S10. Sensitivity or enhanced protection of *Bom* overexpression or loss-of-function mutant lines upon several pathogen challenges

(A) Survival curves of *BomT1*-overexpressing (OE) flies in a wild-type background after *E. faecalis* infection.

(B) Lack-of-sensitivity of *BomT1-Gal4* KI (*$BomT1^{KI}$*) flies to the injection of *M. robertsii* spores

(C-D) Survival curves of *$BomBc1^{V15384}$* (C) and *$BomBc1^{BL65901}$* (D) knock-down RNAi lines after *C. albicans* septic injury.

(E-F) Survival curves of *$BomT2^{\triangle tail}$* (E) and the two *BomT2* null mutants (F) after *C. glabrata* infection.

(G) Survival curves of *$BomBc1^{w1118}$* isoform overexpressing (OE) flies in a *$Bom^{\Delta 55C}$* background after *C. albicans* infection.

Data information: (A-G) Three experiments were performed at different times and each experiment used biological triplicates of 20 flies in parallel. The pooled data were analyzed using Log-Rank test. **: p<0.01; ****: p<0.0001; ns, not significant.

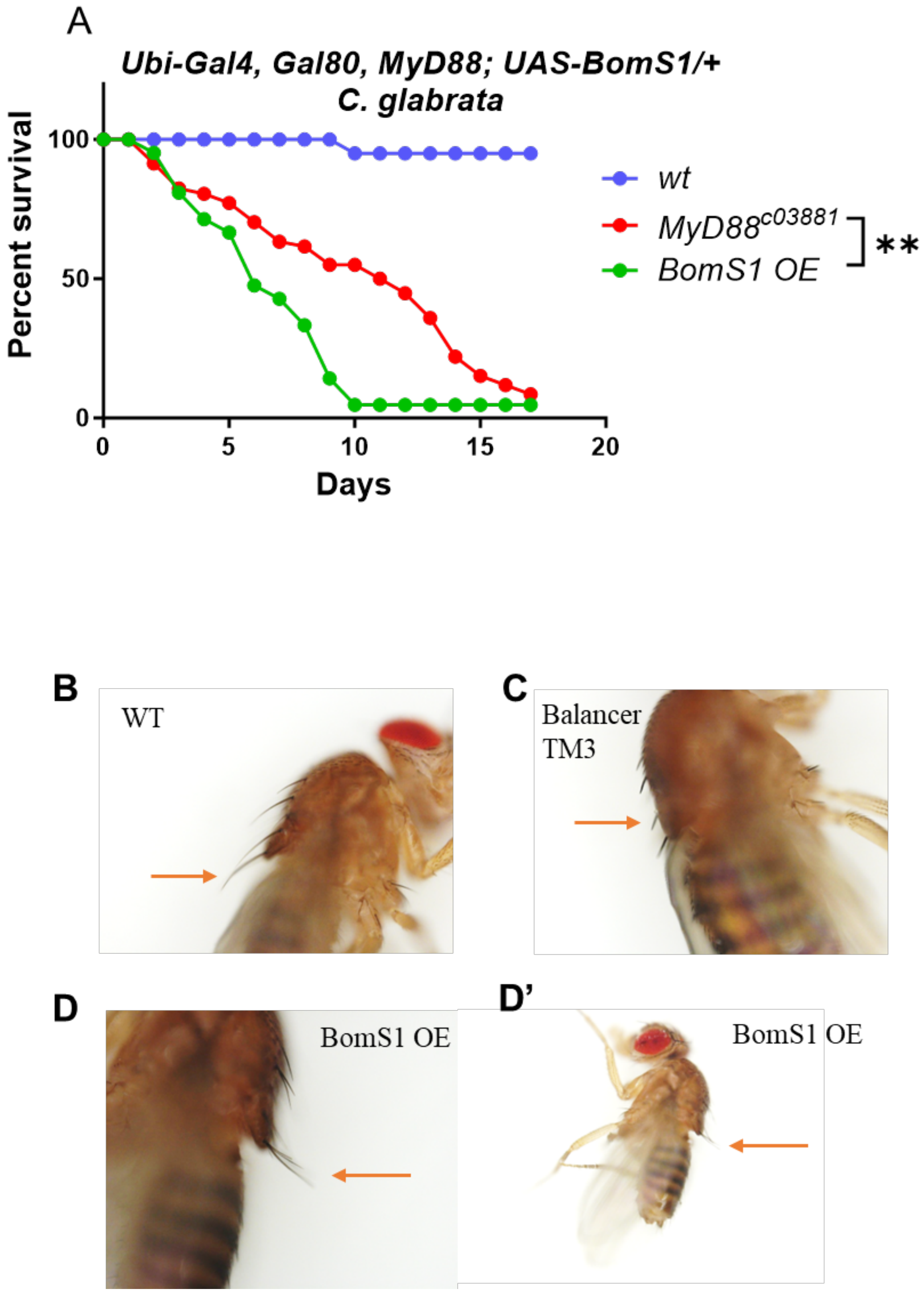

Appendix Figure S11. **The phenotypes of *BomS1*-overexpressing flies in a *MyD88* mutant background**

(A) Survival curves of *BomS1*-overexpressing (OE) flies in a $MyD88^{c03881}$ mutant background after *C. glabrata* infection. The experiment has been performed more than three times.

(B-D) The bristles of flies overexpressing (OE) *BomS1* constantly throughout

development in a wild-type background. Bristles of a wild-type fly (B); stubble bristles from the TM3Sb balancer phenotype (C); stubble-like bristles of *BomS1*-overexpressing flies in a wild-type background (D-D’).

Data information: (A) Three experiments were performed at different times and each experiment used biological triplicates of 20 flies in parallel. The pooled data were analyzed using the Log-Rank test; **: $p<0.01$.

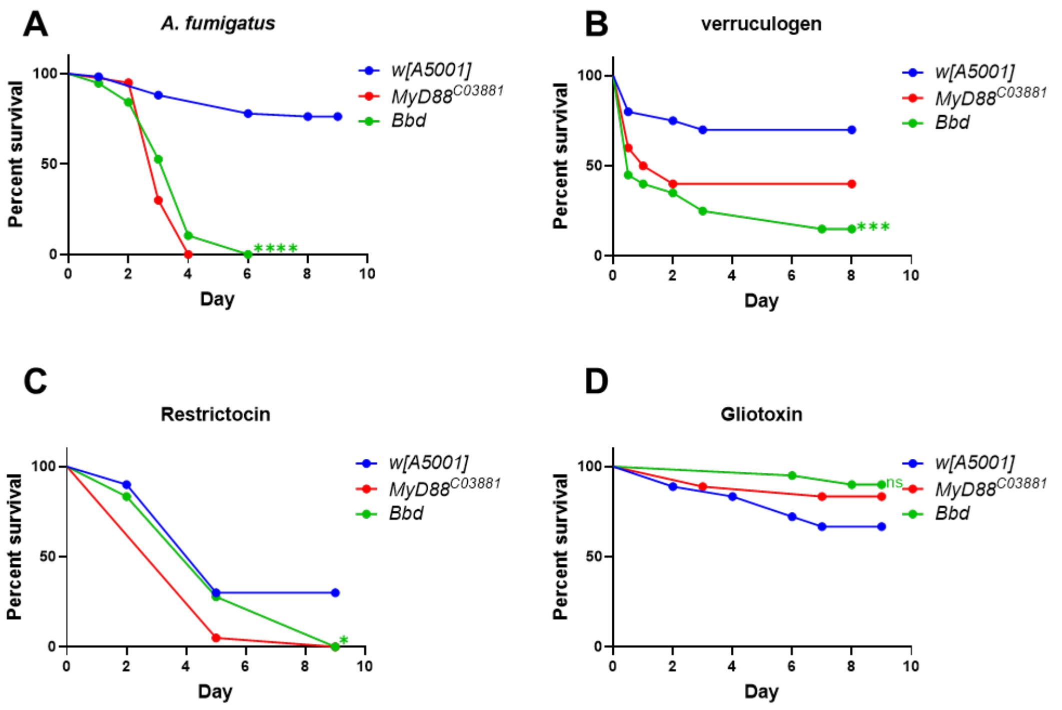

Appendix Figure S12. **Susceptibility of *Bombardier* (*Bbd*) mutants to *A. fumigatus* conidia or to mycotoxin injections.**

(A-D) Survival curves of the *Bombardier* mutants after *A. fumigatus* (A), verruculogen (B)**,** restrictocin (C), and gliotoxin (D) injection.

Data information: (A-D) Three experiments were performed at different times and each experiment used biological triplicates of 20 flies. The pooled data were analyzed using the Log-Rank test; *: p<0.05; **: p<0.01; ***: p<0.001; ****: p<0.0001; ns, no significant difference.

Appendix Tables

Appendix Table S1. Primer sequences for the construction of gRNA plasmids

BomBc1-gSingle-fwd	GCGGCCCGGGTTCGATTCCCGGCCGATGCATTTCGTTGTCCTGGCTTCCCGTTTTAGAGCTAGAAATAGCAAG
BomBc1-gSingle-Rvs	ATTTTAACTTGCTATTTCTAGCTCTAAAACGGTGGCACTTACCTTCACAATGCACCAGCCGGGAATCGAACCC
BomS1-gSingle-fwd	GCGGCCCGGGTTCGATTCCCGGCCGATGCATGATCATCAATGGCGATTGCGTTTTAGAGCTAGAAATAGCAAG
BomS1-gSingle-Rvs	ATTTTAACTTGCTATTTCTAGCTCTAAAACGTGCACTCAGTATCCAAAACTGCACCAGCCGGGAATCGAACCC
BomS4-gSingle-fwd	GCGGCCCGGGTTCGATTCCCGGCCGATGCAGTGAATTGCAATGTTCGTGGGTTTTAGAGCTAGAAATAGCAAG
BomS4-gSingle-Rvs	ATTTTAACTTGCTATTTCTAGCTCTAAAACATGgtgtgtagaacctacaaTGCACCAGCCGGGAATCGAACCC
BomBc2-gSingle-fwd	GCGGCCCGGGTTCGATTCCCGGCCGATGCATGTTTAAACTTATGTGGTGGGTTTTAGAGCTAGAAATAGCAAG
BomBc2-gSingle-Rvs	ATTTTAACTTGCTATTTCTAGCTCTAAAACGACGTTATTGGCGCTTTTGATGCACCAGCCGGGAATCGAACCC
BomS2-gSingle-fwd	GCGGCCCGGGTTCGATTCCCGGCCGATGCATTGAATTCAACTGATGCTCTGTTTTAGAGCTAGAAATAGCAAG
BomS2-gSingle-Rvs	ATTTTAACTTGCTATTTCTAGCTCTAAAACGTCGCCCGATCCAGGAAATGTGCACCAGCCGGGAATCGAACCC
BomS3-gSingle-fwd	GCGGCCCGGGTTCGATTCCCGGCCGATGCAACTCGGGAATTTCTCGATGGGTTTTAGAGCTAGAAATAGCAAG
BomS3-gSingle-Rvs	ATTTTAACTTGCTATTTCTAGCTCTAAAACCGTCTGCAATGTGAGGGCCTTGCACCAGCCGGGAATCGAACCC
BomT2-gSingle-fwd	GCGGCCCGGGTTCGATTCCCGGCCGATGCAATGAAAGCTCTTCAAGTCGCGTTTTAGAGCTAGAAATAGCAAG
BomT2-gSingle-Rvs	ATTTTAACTTGCTATTTCTAGCTCTAAAACGCTGATTTATGGTCTGGAGGTGCACCAGCCGGGAATCGAACCC
BomS5-gSingle-fwd	GCGGCCCGGGTTCGATTCCCGGCCGATGCATCTATGCGGTCTGCTCGCCAGTTTTAGAGCTAGAAATAGCAAG
BomS5-gSingle-Rvs	ATTTTAACTTGCTATTTCTAGCTCTAAAACCGAACATTACAATGACGGCATGCACCAGCCGGGAATCGAACCC
BomS6-gSingle-fwd	GCGGCCCGGGTTCGATTCCCGGCCGATGCACTAGCCAATCCCCTGAGTCCGTTTTAGAGCTAGAAATAGCAAG
BomS6-gSingle-Rvs	ATTTTAACTTGCTATTTCTAGCTCTAAAACctggagtatataaaaatgcgTGCACCAGCCGGGAATCGAACCC

Appendix Table S2. Primers used to identify knock-in/out mutant flies for *BomS2*, *BomT2*, and *BomS5*

	Transgene-specific primer	Sequence
BomS2	*BomS2* KI-F	TGCTGCTCCAAGATTCCAGG
	BomS2 KI-R	AGCTGCAAGGTATTAAAAATACGAA
BomT2	*BomT2* KI-F	TCAGCCTTGCTACTGTATGGT
	BomT2 KI-R	AAGTTCGCAATTTGTCACAAGC
BomS5	*BomS5*-F	AATCAAACCGATTTGCGCGG
	BomS5-R	CTTGAAGCTGTCCTTCCCCG

Appendix Table S3. Primers used for cloning in the expression vector

sequence name	primer sequence
BomS3 primer 1st Fw	AAAAAGCAGGCTTCAACATGAAATTCCTATCACTCGCCTTC
BomS3 primer 1st RvS	AGAAAGCTGGGTCCTANTCANTTAGGCCCTCACATTGCAGACG
BomS5 primer 1st Fw	AAAAAGCAGGCTTCAACATGAAGTGGATGTCCTTGGTCT
BomS5 primer 1st RvS	AGAAAGCTGGGTCTCANCTANTTAGCCTCCGCGAACATTACAA T
BomBc2 primer 1st Fw	AAAAAGCAGGCTTCAACATGAAGAGCCTGACGTTATTG
BomBc2 primer 1st RvS	AGAAAGCTGGGTCCTANTCANTTATCCGCGAATATTGCAACCG
BomS6 primer 1st Fw	AAAAAGCAGGCTTCAACATGAAGCTGCTCTCGATTACTTT
BomS6 primer 1st RvS	AGAAAGCTGGGTCTTANTCANCTAATCGCCGCGTATATTGCAA A
BomT2 primer 1st Fw	AAAAAGCAGGCTTCAACATGAAAGCTCTTCAAGTCG
BomT2 primer 1st RvS	AGAAAGCTGGGTCCTANTCANTTAACTGAATATATGATATATA ATTCC
BomS2 primer 1st Fw	AAAAAGCAGGCTTCAACATGAAGTTCTTCTCAGTCGTCA
BomS2 primer 1st RvS	AGAAAGCTGGGTCTTANTCANCTACTTTCCACCGTGCACATTG
BomS4 primer 1st Fw	AAAAAGCAGGCTTCAACATGCGATTCTTTGCAATCGTCA
BomS4 primer 1st RvS	AGAAAGCTGGGTCTTANTCANCTATTTGCCACCACGAACATTG
BomT1 primer 1st Fw	AAAAAGCAGGCTTCAACATGAAGTGGTTTTCGATTTTGTTTG
BomT1 primer 1st RvS	AGAAAGCTGGGTCCTANTCANTTACCATCTTCGAGTAAATTTG ATTG
BomBc1 primer 1st Fw	AAAAAGCAGGCTTCAAC ATGAAGTGCCTGATTCTGTCCTTTGC
BomBc1 primer 1st RvS	AGAAAGCTGGGTCCTANTCANTTAATCCCCGCCCCCGCTGAT
BomS1 primer 1st Fw	AAAAAGCAGGCTTCAACATGAAATTCTTCTCAGTCGTCACCG

BomS1 primer 1st RvS	AGAAAGCTGGGTCTCANTTANCTACTTGCCACCGTGGACATT
GATEWAY 2nd-Fw *	GGGGACAAGTTTGTACAAAAAAGCAGGCT
GATEWAY 2nd-Rv *	GGGGACCACTTTGTACAAGAAAGCTGGGT
HL1-F **	TGGACCTGCGGGGTTAATTTACC
attP-R **	TTGTGTCATGTCGGCGACCCTACG
hsp-GW-F ***	GCAACTACTGAAATCTGCCAAG
HL1-R ***	AATTAACCCCGCAGGTCCACCGG

Note: *, primer for second round PCR to build attB sequence;

**, primer for barcode amplification;

***, amplify ORF of interesting gene in transgenic fly

Appendix Table S4. Primers used for quantitative RT-PCR

primer name	RT-qPCR primer	primer efficiency
RpL32-Fq	GACGCTTCAAGGGACAGTATCTG	96.80%
RpL32-Rq	AAACGCGGTTCTGCATGAG	
BomS1-Fq	CAATGCTGTTCCACTGTCGC	100.90%
BomS1-Rq	CGTGGACATTGCACACCCTG	
BomBc1-Fq	TTTCGTTGTCCTGGCTTCCC	96.40%
BomBc1-Rq	CCGACTACGACGTTTTCCGC	
BomBc2-Fq	AGAGCCTGACGTTATTGGCG	96.30%
BomBc2-Rq	TGGGAGCAGTTTACGCACTT	
BomS2-Fq	AGTCGTCACCGTCTTTGTGTT	100.90%
BomS2-Rq	CAGTATTTGCAGTCCCCGTTG	
BomS3-Fq	TCACTCGCCTTCGTTTTGGG	103.80%
BomS3-Rq	TTAGGCCCTCACATTGCAGAC	
BomT2-Fq	TAATGCTACGCCGGGACAAG	98.20%
BomT2-Rq	ATGGCTCCAGATGTGAGTGTG	
BomS4-Fq	GTTGTCACCCGATCCAGGAA	94.80%
BomS4-Rq	ATTTGCCACCACGAACATTGC	
BomT1-Fq	TCCCAGGATTACCATTCGCA	95.40%
BomT1-Rq	GAGTTTTTGGTGGCGCGTG	
BomS5-Fq	GGTCTTTCTATGCGGTCTGCT	91.40%
BomS5-Rq	TAGCCTCCGCGAACATTACA	
BomS6-Fq	GCTAGTGCCAATCCCCTGAG	104.50%
BomS6-Rq	TCGCCGCGTATATTGCAAAC	
