# Supplementary material for: Distinct Bomanins at the Drosophila 55C locus function in resistance and resilience to infections
Source: EMBO Rep. 2026 Jan 9;27(3):629–53. doi: 10.1038/s44319-025-00559-6 (PMC12894722; doi:10.1038/s44319-025-00559-6)
Supplement: Supplementary file 10 — Expanded View Figures [file 44319_2025_559_MOESM10_ESM.pdf]

# Expanded View Figures

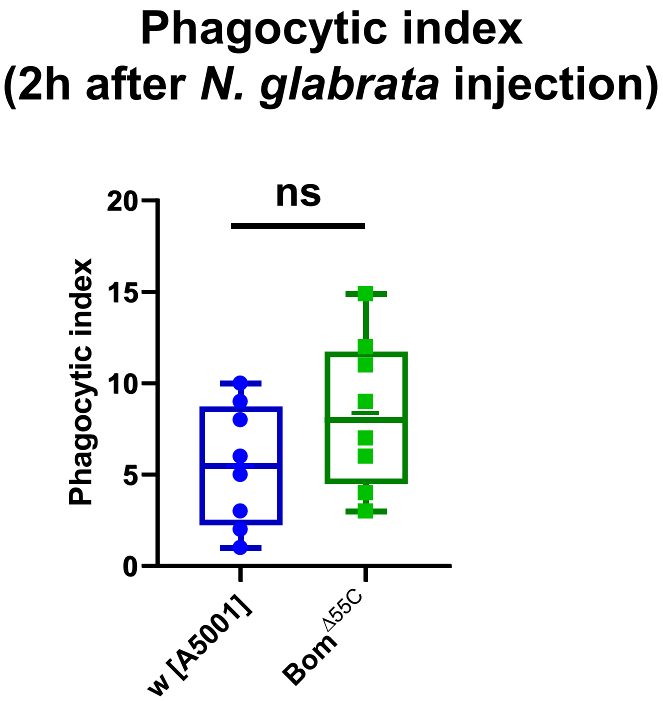


**Figure EV1.** ***Bomanins* at *55C* locus are not involved in phagocytosis.**

6000 live yeasts *N. glabrata* cells were injected into *w* [A5001] wild-type flies and $Bom^{\Delta 55C}$ flies and their phagocytic index was monitored 2 h after injection. Data information: Mann–Whitney test. ns: not significant. The middle bar of the boxplot represents the median and the upper and lower limits of the box indicate respectively the first and third quartiles; the whiskers define the minima and maxima; $Bom^{\Delta 55C}$ flies: $P = 0.15$. Source data are available online for this figure.

© The Author(s) 

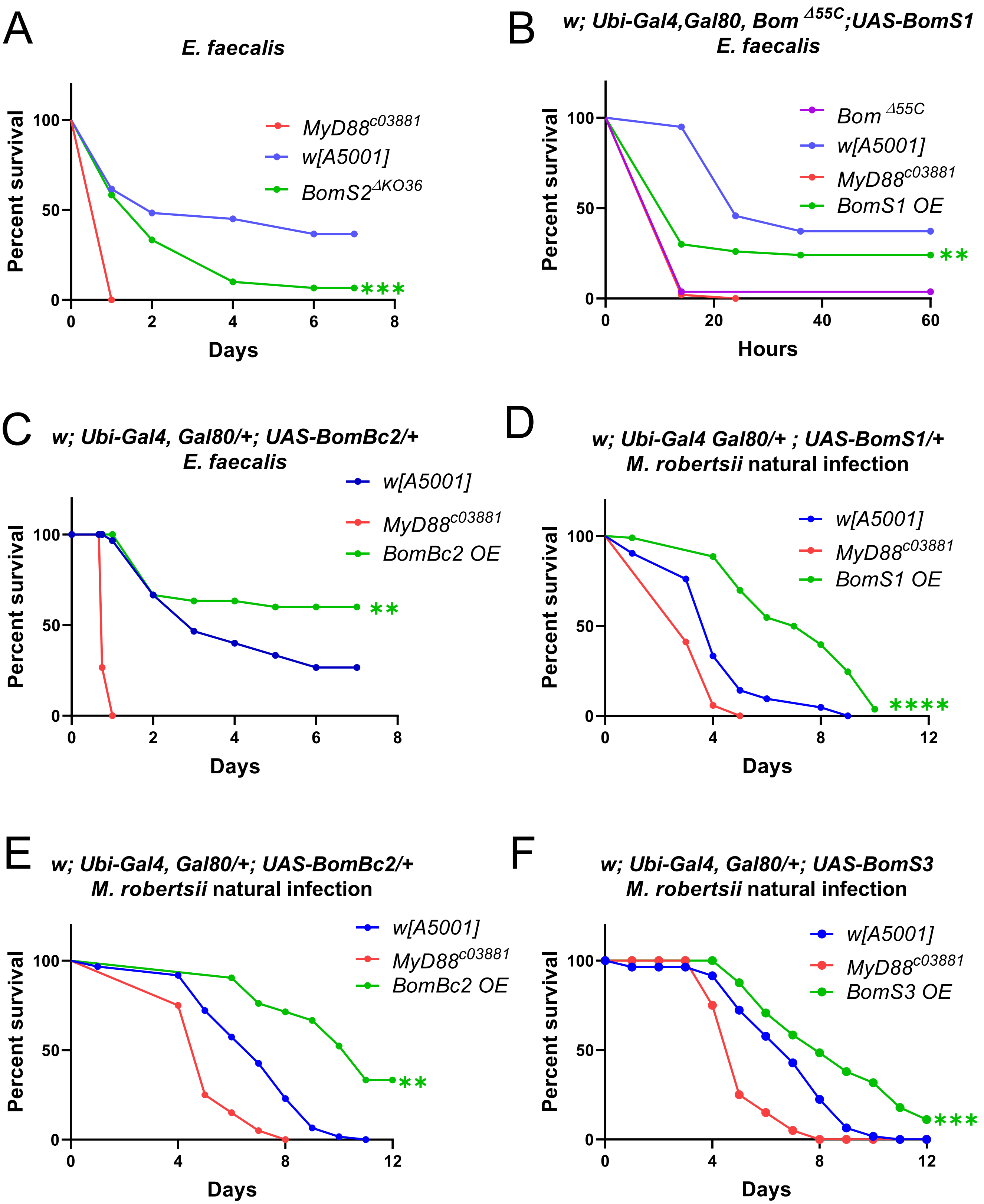
A
E. faecalis
Percent survival
Days
MyD88c03881
w[A5001]
BomS2ΔKO36
***
B
w; Ubi-Gal4,Gal80, Bom Δ55C;UAS-BomS1
E. faecalis
Percent survival
Hours
Bom Δ55C
w[A5001]
MyD88c03881
BomS1 OE
**
C
w; Ubi-Gal4, Gal80/+; UAS-BomBc2/+
E. faecalis
w[A5001]
MyD88c03881
BomBc2 OE
Percent survival
Days
**
D
w; Ubi-Gal4 Gal80/+ ; UAS-BomS1/+
M. robertsii natural infection
w[A5001]
MyD88c03881
BomS1 OE
Percent survival
Days
****
E
w; Ubi-Gal4, Gal80/+; UAS-BomBc2/+
M. robertsii natural infection
w[A5001]
MyD88c03881
BomBc2 OE
Percent survival
Days
**
F
w; Ubi-Gal4, Gal80/+; UAS-BomS3
M. robertsii natural infection
w[A5001]
MyD88c03881
BomS3 OE
Percent survival
Days
***

 © The Author(s)

**Figure EV2. Overexpression of *BomS1, BomBc2, or BomS3* in wild-type or *Bom*$^{\Delta55C}$ background enhances resistance to *E. faecalis* or *M. robertsii.***

(**A**) Survival curves of *BomS2*$^{\Delta KO36}$ null mutant flies after *E. faecalis* infection. (**B**) Survival curves of *BomS1*-overexpressing (OE) flies in a *Bom*$^{\Delta55C}$ background after *E. faecalis* injection. (**C**) Survival experiments of *BomBc2*-overexpression (OE) flies in a wild-type background after *E. faecalis* injection. (**D–F**) Survival curves of *BomS1* (**D**), *BomBc2* (**E**), *BomS3* (**F**) overexpressing (OE) flies in a wild-type background after *M. robertsii* natural infection. Data information: In (**A**–**F**) three experiments were performed at different times and each experiment used biological triplicates of 20 flies in parallel. The pooled data were analyzed between infected-mutant and - *w* [A5001] fly, except for (**B**) that compared *BomS1 OE* in a *Bom*$^{\Delta55C}$ background to *Bom*$^{\Delta55C}$, using the Log-Rank test; ns, no significant difference. (**A**) *BomS2*$^{\Delta KO36}$ flies: $P = 0.0002$; (**B**) BomS1 OE: $P = 0.0013$; (**C**) BomBc2 OE: $P = 0.0007$; (**D**) BomS1 OE: $P < 0.0001$; (**E**) BomBc2 OE: $P = 0.0025$; (**F**) BomS3 OE: $P = 0.0009$. Source data are available online for this figure.

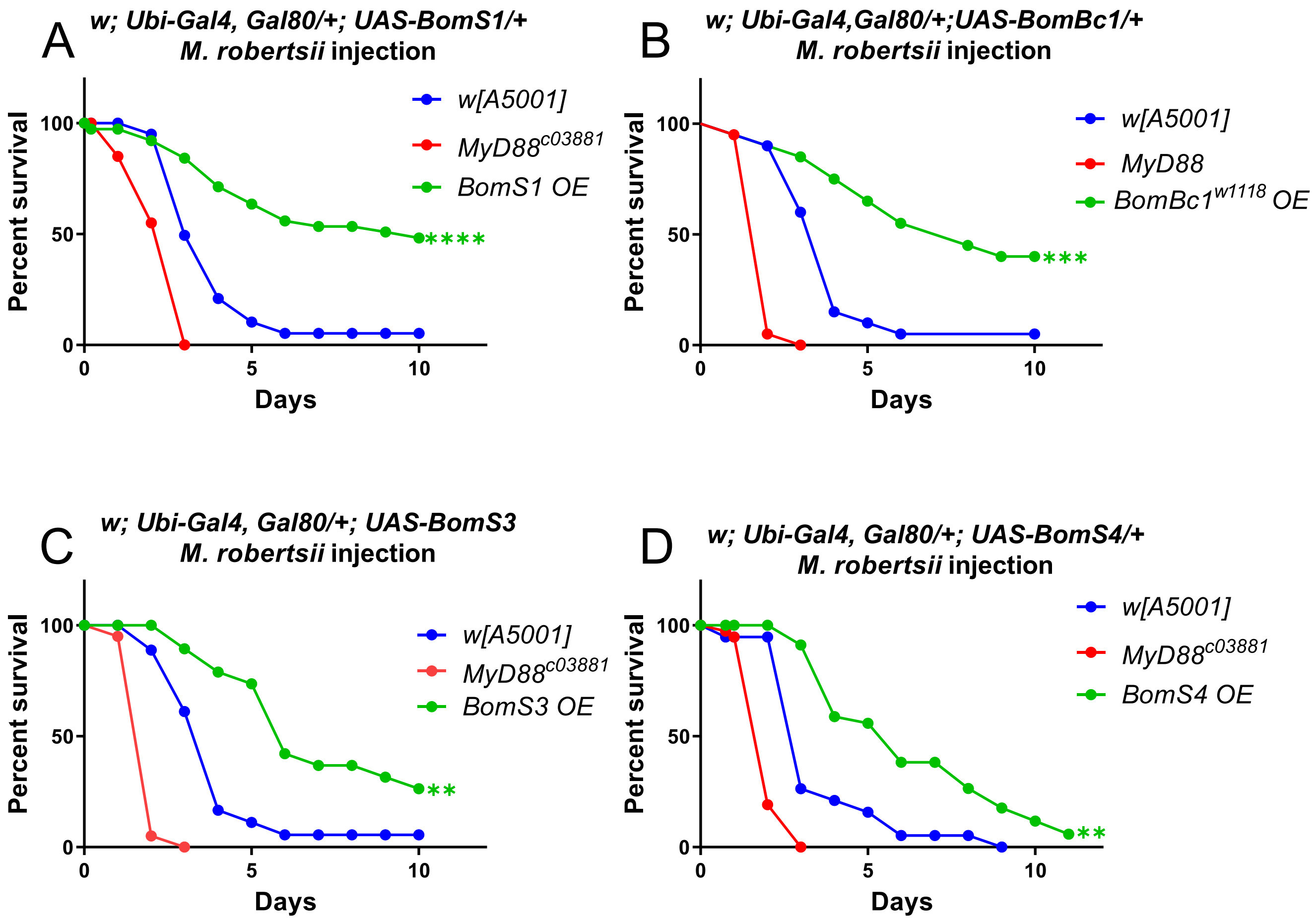


**Figure EV3. Overexpression of *BomBc1, BomS1, BomS3*, and *BomS4* in a wild-type genetic background can protect them from injected *M. robertsii* spores.**

(**A–D**) Survival curves of *BomBc1* (**A**), *BomS1* (**B**), *BomS3* (**C**), and *BomS4* (**D**)-overexpressing (OE) flies in a wild-type background after *M. robertsii* injection. Data information: three experiments were performed at different times and each experiment used biological triplicates of 20 flies in parallel. The pooled data were analyzed between infected-mutant and - *w* [A5001] fly using the Log-Rank test; ns, no significant difference. (**A**) BomS1 OE: $P < 0.0001$; (**B**) BomBc1w1118 OE: $P = 0.0003$; (**C**) BomS3 OE: $P = 0.0029$; (**D**) BomS4 OE: $P = 0.0072$. Source data are available online for this figure.

 © The Author(s)

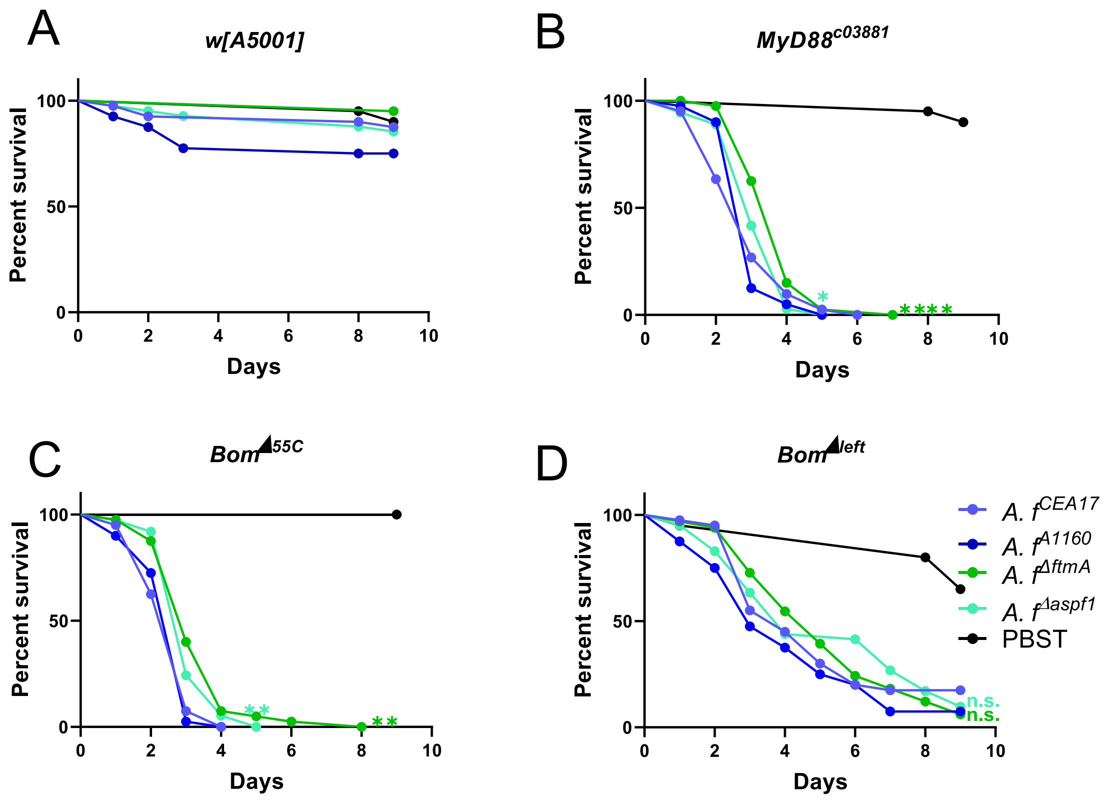


**Figure EV4. Mycotoxins mutants of *A. fumigatus* are as virulent as wild-type fungi when injected into $Bom^{\Delta left}$ flies.**

(**A–D**) Survival curves of *w*[A5001] (**A**), $MyD88^{c03881}$ (**B**), $Bom^{\Delta 55C}$ (**C**), and $Bom^{\Delta left}$ (**D**) flies to *A. fumigatus*$^{\Delta ftmA}$ (fumitremorgin/verruculogen pathway inactivated), *A. fumigatus*$^{\Delta aspf1}$ (restrictocin mutant), and *A. fumigatus* genetic background controls (*A. fumigatus*$^{CEA17}$ or *A. fumigatus*$^{A1160}$) injection. Data information: three experiments were performed at different times and each experiment used biological triplicates of 20 flies in parallel. The pooled data were analyzed between mycotoxin mutant infected flies and wild-type *A. fumigatus* control-infected flies using the Log-Rank test; ns, no significant difference. (**A**) *A. fumigatus*$^{\Delta ftmA}$: $P = 0.22$, *A. fumigatus*$^{\Delta aspf1}$: $P = 0.86$; (**B**) *A. fumigatus*$^{\Delta ftmA}$: $P < 0.0001$, *A. fumigatus*$^{\Delta aspf1}$: $P = 0.047$; (**C**) *A. fumigatus*$^{\Delta ftmA}$: $P = 0.0013$; *A. fumigatus*$^{\Delta ftmA}$: $P = 0.0011$; (**C**) *A. fumigatus*$^{\Delta ftmA}$: $P = 0.93$; *A. fumigatus*$^{\Delta ftmA}$: $P = 0.99$. Source data are available online for this figure.
