# Supplementary material for: Distinct Bomanins at the Drosophila 55C locus function in resistance and resilience to infections
Source: EMBO Rep. 2026 Jan 9;27(3):629–53. doi: 10.1038/s44319-025-00559-6 (PMC12894722; doi:10.1038/s44319-025-00559-6)
Supplement: Supplementary file 4 — Source data Fig. 2 [file 44319_2025_559_MOESM4_ESM.zip › Source data/figure 2/Figure 2C/RWG7110 20141 CRISPR Design Repoet_BomT1-Gal4 knockout.pdf]

CRISPR Design Report

**Report No.:**	RWG7110	**Date:**	2020.06.24
**Reporter:**	Dr. Pei-Tseng Lee	**E-mail:**	peitseng.lee@wellgenetics.com

****

Gene: *BomT1/CG43202* **Case No.:** 20141

Project: making a Gal4 reporter knockout of *BomT1* by deleting entire CDS and knocking in Gal4 and a floxed selection marker to facilitate genetic screening

Method: CRISPR/Cas9-mediated genome editing by homology-dependent repair (HDR) using two guide RNA(s) and a dsDNA plasmid donor

Gene and Method

Gene: *BomT1/CG43202*

Location: 2R:18,381,393..18,381,844 [-]

Method: CRISPR/Cas9-mediated genome editing by homology-dependent repair (HDR) using two guide RNAs and a dsDNA plasmid donor

Knock-in cassette: Gal4-3xP3-RFP

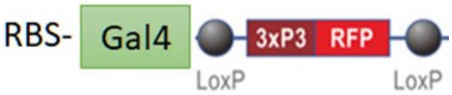

Injection strain: *w[1118]*

Genome Editing:

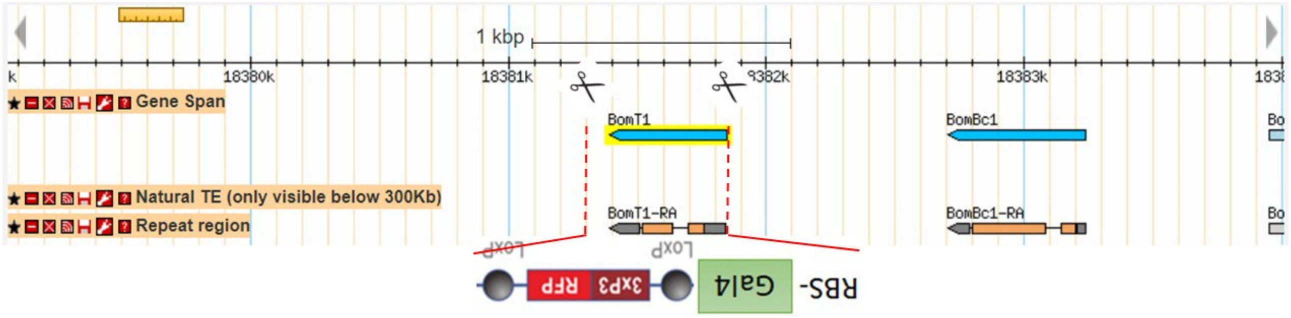

(1) A 545-bp fragment of *BomT1* (-091 nt to +454 nt from ATG of *BomT1*) will be deleted and be replaced with cassette Gal4-3XP3-RFP.

(2) Cassette Gal4-3xP3-RFP contains ribosome binding sequence (RBS), Gal4, SV40 3'UTR and 3xP3-RFP as a selection marker.

(3) The selection marker 3xP3-RFP contains loxP site, 3x Pax3 promoter, RFP, alpha-Tubulin 3'UTR and loxP site. It facilitates the genetic screening and can be flipped out by Cre recombinase.

WELLGENETICS

CRISPR Target Sites

Guide RNA Quality Instruction

Guide RNA quality is shown in color dots for your references.

	Distance	Off target	GC Content	T #
(green) Strong site	<50bp	0	45%-70%	≦1
(yellow) Weak site	50-100bp	Potential off targets on untargeted chromosomes	40%, 75%	2
(red) Bad site	>100bp	Potential off targets on targeted chromosomes	< 40%, >75%	≧3

1. The distance from knockin site or deletion breakpoint to cutting site of Cas9.
2. Predicted off targets on other chromosomes are indicated.
3. GC content 45% to 70% of target sequence provides the best efficiency.
4. High thymine numbers at 17th to 20th nucleotide of the guide RNA target site may decrease the efficiency.

gRNA 1 (upstream)

CRISPR Target Site [PAM]: ATCTCGACGGTGAATAACAC[GGG]

CRISPR Target Strand in the Genome: plus

Cutting Site: -91 nt from ATG of *BomT1*

Distance: +0 nt from upstream breakpoint to cutting site of Cas9 (green)

GC Content: 45% (green)

T number at 17th to 20th Nucleotides: 0 (green)

Off target: 0 (green)

Guide RNA Primers:

Sense oligo 5'- CTTCGATCTCGACGGTGAATAACAC

Antisense oligo 5'- AAACGTGTTATTCACCGTCGAGATC

PAM mutation: not required

Upstream Homology Arm: 1,016bp, the -1,107 nt to -92 nt from ATG of *BomT1*

Forward Oligo 5'- TGGTTACAGTGGTCGTCTGC

Reverse Oligo 5'- CACGGGAGTAACAGAAAATACCC

Downstream Homology Arm: same as below

gRNA 2 (downstream)

WELLGENETICS

CRISPR Target Site [PAM]: AGTACACTGCTGAAGCAGTC[AGG]

CRISPR Target Strand in the Genome: plus

Cutting Site: +203 nt from Stop Codon of *BomT1*

Distance: +0 nt from downstream breakpoint to cutting site of Cas9 ●

GC Content: 50% ●

T number at 17th to 20th Nucleotides: 1 ●

Off target: 0 ●

Guide RNA Primers:

Sense oligo 5’- CTTCGAGTACACTGCTGAAGCAGTC

Antisense oligo 5’- AAACGACTGCTTCAGCAGTGTACTC

PAM mutation: not required

Upstream Homology Arm: same as above

Downstream Homology Arm: 1,026bp, the +204 nt to +1,229 nt from Stop Codon of *BomT1*

Forward Oligo: 5’- TGCTTCAGCAGTGTACTAAATTAA

Reverse Oligo: 5’- TGTGTGCAATACGTTTTACTGG

Donor Design

Annotation

NNNNN: Homology Arm / Coding Region

NNNNN: Homology Arm / UTR

nnnnn: Homology Arm/ Intron

nnnnn: Homology Arm/ Intergenic Region

nnnnn: gRNA target sequence

nnnnnn: RE site

NNNNN: Gal4

nnnnn: SV40 3'UTR

nnnnn: LoxP

nnnnn: 3xP3 Promoter

NNNNN: RFP

nnnnn: alpha Tubulin 3'UTR

Sequence

TGGTTACAGTGGTCGTCTGCCAGGTGGCACTTACCTTCACAATAAGGATTGCGTGGGCTGCAGCATCAGCGG
GGGCGGGGATTAACGAAATTATATTGTGATTTGTACATAAAATACCTCGCATTTTTTAATTTTCGGGCATAATTTA
TAATTCTTTAAAAAATCTGaaaatttggtatagttactttatgtcatattttatagaatcttattggttcagacctttataaatgtcctttataa
attttgataaaattaattttccaactgtttttgagtttcgtagagacaaacatttttaattaaaaagtaaaattatataagagctattaagtaatca
aagctaataaaaaagttataaatcaattagaaaatcaaaaagaccctcgtttaatacgcttattaaacatatgatggcattttattaccaattta
aaattttaatttttccttttcatttctttttatcaaagcattgtttattggtcggaaaaaaaaagttttgtttgcgcaatatacaagtttacatactttac
ttccatagaattttattaatatttgcggtattcgaccataattgaatacatacatgcactgaaaatactttttacgacaatattcatttttcaatttaa
atgaaattctacaaaaatattaagaacttcaaatgtgtgttttaaataaagtagttcgaaaattaactttaacagatacttagtatagtttttcaag
taatagagcttcatattacttatgattattacacaactcgaaaataatatgcattgcatatctgaaatattccccctacgtcatattactcttatcac
aactataacttagaatactacttaaataataagctgatttagttgaagcttttgagatgcttttgagatgcactgcctctgagtcaaacgttcagtt
gagtggtatacgttatcaagagcgatggggcttttggaaaacccaaagatctataaaatattctagggtattttctgttactcccgtg
agatctgaagcaagcctcaatcaaaaccATGAAGCTGCTGAGTAGTATTGAACAAGCGTGTGACATATGCCGACTTAAG
AAGCTGAAGTGCAGCAAGGAAAAGCCGAAGTGCGCCAAATGTCTTAAGAACAATTGGGAGTGCCGGTACTC
GCCAAAGACGAAGCGAAGCCCTTTGACACGCGCTCATTTGACTGAAGTGGAGAGTCGCCTGGAGCGCCTTG
AGCAGTTGTTCCTCCTCATTTTTCCCCGTGAAGACCTCGACATGATACTGAAGATGGATAGTCTCCAAGATATTA
AGGCCCTGCTCACTGGCCTGTTTGTTCAGGATAACGTCAACAAGGATGCCGTGACGGACCGCCTTGCCTCGG
TGGAGACAGATATGCCACTTACCTTGCGGCAGCATCGCATCTCGGCTACCAGCAGTAGTGAAGAAAGTTCCAA
TAAGGGTCAACGGCAACTGACGGTATCGATCGACAGTGCGGCCCACCACGACAACAGCACCATTCCACTGGA
CTTCATGCCTCGTGACGCACTGCATGGATTTGACTGGAGCGAGGAGGATGATATGAGCGACGGACTCCCATTC

CTGAAGACCGACCCCAATAACAATGGTTTCTTTGGCGACGGCAGCCTGCTGTGTATCCTGCGAAGTATTGGTT
TTAAGCCGGAAAATTACACGAACAGTAATGTCAATCGTCTTCCCACAATGATCACCGACCGTTACACGCTGGCC
AGTCGCAGTACTACAAGCCGCCTGCTGCAAAGCTATCTTAACAACTTCCACCCCTACTGCCCCATAGTGCATAG
TCCTACTCTCATGATGCTTTACAACAATCAGATTGAGATAGCATCGAAGGACCAGTGGCAGATCCTCTTCAACT
GCATTCTCGCCATCGGCGCTTGGTGTATTGAAGGCGAGAGCACAGATATCGACGTGTTTTACTATCAAAACGC
CAAGTCGCACCTCACCTCGAAGGTCTTCGAAAGTGGAAGCATTATCCTCGTGACAGCCCTCCATCTGCTGAGT
CGTTATACCCAGTGGCGTCAGAAAACTAATACATCCTACAACTTTCATTCGTTCAGCATTCGGATGGCGATTAGC
CTGGGACTTAATCGTGACCTCCCAAGTAGCTTCAGCGATTCGTCGATCCTGGAACAGCGCCGGAGGATTTGGT
GGAGTGTGTACAGCTGGGAGATCCAGCTGTCCCTCTTGTACGGACGCAGCATTCAGCTGAGTCAGAACACGA
TTTCCTTCCCGTCCAGCGTGGACGACGTTCAACGGACAACTACGGGCCCGACCATCTACCACGGTATCATTGA
GACGGCCCGCTTGCTTCAAGTGTTTACGAAGATTTATGAGCTGGATAAGACAGTGACCGCGGAGAAGAGTCC
GATCTGCGCCAAGAAATGCCTGATGATCTGCAATGAGATAGAGGAGGTGAGCCGCCAGGCACCCAAGTTCCT
GCAGATGGACATAAGCACTACGGCGCTGACTAATCTCCTCAAAGAACACCCCTGGCTGAGTTTCACCCGCTTC
GAGCTGAAGTGGAAACAGCTGAGTCTGATTATATACGTTTTGCGGGACTTTTTCACAAATTTTACGCAGAAGA
AGTCGCAGCTTGAGCAAGATCAGAACGACCATCAGTCCTACGAGGTAAAGAGGTGCAGCATAATGCTCTCCG
ACGCAGCCCAGCGCACCGTGATGTCGGTGTCGTCCTATATGGACAACCATAATGTGACCCCTTATTTCGCCTGG
AATTGCTCCTACTATTTGTTCAACGCCGTGTTGGTCCCAATCAAGACTCTTCTGAGCAACTCCAAATCGAACGC
CGAGAATAACGAGACAGCACAGCTCTTGCAGCAAATTAACACCGTGTTGATGCTGTTGAAGAAACTCGCTAC
ATTCAAGATTCAGACTTGCGAAAAGTACATCCAGGTGTTGGAAGAGGTGTGCGCCCCATTCCTTCTTTCGCAA
TGCGCAATTCCCCTCCCGCACATAAGTTACAATAATTCCAACGGCTCCGCTATCAAGAATATCGTCGGCTCCGCT
ACAATCGCGCAGTATCCCACGCTGCCTGAGGAAAATGTTAACAACATATCGGTGAAGTATGTAAGTCCAGGCA
GCGTCGGCCCGAGCCCTGTGCCGCTGAAAAGCGGAGCCTCCTTCAGCGATCTGGTCAAGCTGCTCAGCAACC
GCCCACCATCCCGCAACTCCCCTGTCACTATACCCCGATCGACCCCGTCGCATCGTAGCGTTACCCCATTCCTGG
GACAACAGCAGCAACTGCAGAGTCTGGTGCCACTTACGCCCTCGGCATTGTTTGGCGGAGCCAACTTCAACC
AGAGTGGTAACATTGCGGACAGCTCCTTGAGCTTCACGTTTACCAACAGCTCCAACGGTCCAAACTTGATCAC
GACTCAGACAAACTCCCAGGCATTGTCCCAGCCCATTGCCAGCTCGAATGTGCATGATAACTTTATGAATAATG
AAATTACTGCCTCCAAGATTGACGACGGCAACAACAGCAAGCCCCTTTCCCCAGGCTGGACTGACCAGACGG
CCTACAATGCTTTCGGTATCACCACTGGCATGTTCAACACCACAACGATGGACGACGTATATAACTATCTTTTTG
ATGATGAGGATACTCCACCGAACCCAAAGAAGGAGTAG**aagcttcctagg**gatctttgtgaaggaaccttacttctgtggtgt
gacataattggacaaactacctacagagatttaaagctctaaggtaaatataaaatttttaagtgtataatgtgttaaactactgattctaattgt
ttgtgtattttagattccaacctatggaactgatgaatgggagcagtggtggaatgcctttaatgaggaaaacctgttttgctcagaagaaatgc
catctagtgatgatgaggctactgctgactctcaacattctactcctccaaaaaagaagagaaaggtagaagaccccaaggactttccttcaga
attgctaagtttttgagtcatgctgtgtttagtaatagaactcttgcttgctttgctatttacaccacaaaggaaaaagctgcactgctatacaag
aaaattatggaaaaatatttgatgtatagtgccttgactagagatcataatcagccataccacatttgtagaggttttacttgctttaaaaaacct
cccacacctccccctgaacctgaaacataaaatgaatgcaattgttgttgttaacttgtttattgcagcttataatggttacaaataaagcaatag

catcacaaatttcacaaataaagcatttttttcactgcattctagttgtggtttgtccaaactcatcaatgtatcttatcatgtctaccggtgatctat
aacttcgtataatgtatgctatacgaagttat**ggtacc**ggatctaattcaattagagactaattcaattagagctaattcaattaggatccaagct
tatcgatttcgaaccctcgaccgccggagtataaatagaggcgcttcgtctacggagcgacaattcaattcaaacaagcaaagtgaacacgtc
gctaagcgaaagctaagcaaataaacaagcgcagctgaacaagctaaacaatcgggcggccgcactagagccggtcgccaccATGAGG
TCTTCCAAGAATGTTATCAAGGAGTTCATGAGGTTTAAGGTTCGCATGGAAGGAACGGTCAATGGGCACGAG
TTTGAAATAGAAGGCGAAGGAGAGGGGAGGCCATACGAAGGCCACAATACCGTAAAGCTTAAGGTAACCAA
GGGGGGACCTTTGCCATTTGCTTGGGATATTTTGTCACCACAATTTCAGTATGGAAGCAAGGTATATGTCAAGC
ACCCTGCCGACATACCAGACTATAAAAAGCTGTCATTTCCTGAAGGATTTAAATGGGAAAGGGTCATGAACTT
TGAAGACGGTGGCGTCGTTACTGTAACCCAGGATTCCAGTTTGCAGGATGGCTGTTTCATCTACAAGGTCAAG
TTCATTGGCGTGAACTTTCCTTCCGATGGACCTGTTATGCAAAAGAAGACAATGGGCTGGGAAGCCAGCACT
GAGCGTTTGTATCCTCGTGATGGCGTGTTGAAAGGAGAGATTCATAAGGCTCTGAAGCTGAAAGACGGTGGT
CATTACCTAGTTGAATTCAAAAGTATTTACATGGCAAAGAAGCCTGTGCAGCTACCAGGGTACTACTATGTTGA
CTCCAAACTGGATATAACAAGCCACAACGAAGACTATACAATCGTTGAGCAGTATGAAAGAACCGAGGGACG
CCACCATCTGTTCCTTTAGcggccatcgaattcgagctcgcccactaagcgtcgcgccacttcaacgctcgatgggagcgtcattggtgg
gcggggtaaccgtcgaaatcagtgtttacgcttccaatcgcaacaaaaaattcactgcaacactgaaaagcatacgaaaacgatgaagattgt
acgagaaaccataaagtattttatccacaaagacacgtatagcagaaaagccaagttaactcggcgataagttgtgtacacaagaataaaat
cggccagattcagtgttgtcagaaataagaaaccccactatgtttttctttgccttttctttctcccagcgatcattcatttcgtggtgaaagaacg
gggtcattgcacggagtttcgactgcgggaaagcagagctgccgttcacttcgtctataattagcgctttctattttccccgattcgggccgctgct
gcgcttttccgcctgctgtttgtggcaagtgtagcagcaggctgtgcacgcagtgtggcatgcacttggctttccaccgttggtatcgattctctgg
gacgatgagtcattcctttcggggccacagcataatcgttgccagctcaccgaaatggtgacttcatttcttaactgccgtcaagcatgcgattgt
acatacatacatatttatatatgtacatatttatgtgactatggtaggtcgatataatagcaatcaacgcaagcaaatgtgtcagtcctgcttaca
ggaacgattctatttagtaattttcgttgtataaagtaattatgtatgtatgtaagccccataaatctgaaacaattaggcaaaaccatgcgaagc
t**ctgcag**ataacttcgtataatgtatgctatacgaagttat**gctagc**

tgcttcagcagtgtactaaattaataatagccaaattgcaatgttctcgtaaatttgtcgtaaactcaagaccggatcgtaaatcatttgtggggc
ccgaggaaagttccgttttggcttctgttttatgcctcattggtggaaatatttcaacaaaaaggcataaaaattgccatcatatttggctataccc
gccaccaggccgtcgaacacgtgtttttctcgttttccttgcggcaaaggcaaaggcattgccacataccgcgctttccgcgtgtcctgtgacccg
tgtcctttgagagatgttttattgccaaagaaaaccgagcgaaggcaaaccgctgtgcgcctgtaaatgactcctcaggcacctttatattgact
cgtaaaattgcgacatttagttgtatgtgagggggggggtggccaatccaattcaatccctaccagacattttcggcctgcttttcgaggtagaagt
cacgggtttccgaggggcttctcagagctttaaatttgatttttatttgccccaaccgttgttaccggcaaacgatattttttttatcgcacacaaa
aactcttcgtccttctagtctctgatttaattcagcggaaattgagttttggccgggtagaaaatggtggaaaacaatgtcaagtggagcatgaa
aggaatcaaacacttgatagatttttccacttgccactccctttttgcctgtaatatgaacctttgtgcagcaactttgattaatttcgttttcgaggt
atgaagaatttaattgaatttttcaagtgtgctgccgataaggactcaatttttaataattacattcatctcgttacgtttgccgacggcaaattgcg
catacgcacagttgcacatataaattttcattttttttttcatttttataccgagtgaaagaggctttgcctgttatttgtatttccatcatgattttttt
gaatatttatagcgatcagaaagtgaaaaatgccagtaaaacgtattgcacaca
